# Mathematical model for metabolic neuro-hemodynamic coupling

**DOI:** 10.1186/1471-2202-12-S1-P170

**Published:** 2011-07-18

**Authors:** Hyuk Kang, Sun Mi Park, Dong-Uk Hwang, Daeshik Kim

**Affiliations:** 1 Computational Neuroscience Team, National Institute for Mathematical Sciences, Daejeon, 305-811, Republic of Korea; 2 Electrical Engineering, Korea Advanced Institute of Science & Technology, Daejeon, 305-701, Republic of Korea

## 

Via advances of measuring techniques, neuronal activities have been estimated in more detail. Especially, by using the fMRI method, the BOLD (Blood Oxygen Level Dependent) Signal, which is defined by the hemoglobin concentration in the blood vessel, shows various activities of neurons near the vessel. Such BOLD signals are described by the hemoglobin level, vessel volume, and blood vessel flow, which are determined via the Balloon model [1]. Otherwise, the oxygen and the glucose, which have the important role for the neuronal activity, are transported from the blood vessel to the neuron directly or indirectly through the astrocyte. So, in order to explain the contribution of neuronal activity to the alteration of BOLD signal, it has been needed to consider the metabolic pathway which describes the oxygen and glucose consumption process of the neuron and the astrocyte [2]. Here, for describing the relationship between the blood oxygen level and the neuronal activity, we introduce the mathematical model for the interactions among a neuron, astrocyte, and capillary. Especially, we adapt the two compartment neuron model, which constitutes somatic and dendritic parts, for observing the alteration of the BOLD signal depending on the various neuronal spike types [3], and investigate the sodium, ATP, lactate, glucose, oxygen concentrations in the neuron and astrocyte
